# Vampire Venom: Vasodilatory Mechanisms of Vampire Bat (*Desmodus rotundus*) Blood Feeding

**DOI:** 10.3390/toxins11010026

**Published:** 2019-01-08

**Authors:** Rahini Kakumanu, Wayne C. Hodgson, Ravina Ravi, Alejandro Alagon, Richard J. Harris, Andreas Brust, Paul F. Alewood, Barbara K. Kemp-Harper, Bryan G. Fry

**Affiliations:** 1 Department of Pharmacology, Biomedicine Discovery Institute, Faculty of Medicine, Nursing & Health Sciences, Monash University, Clayton, Victoria 3800, Australia; rahini.r.ragavan@monash.edu (R.K.); wayne.hodgson@monash.edu (W.C.H.); ravina.ravi@monash.edu (R.R.); barbara.kemp@monash.edu (B.K.K.-H.); 2 Departamento de Medicina Molecular y Bioprocesos, Instituto de Biotecnología, Universidad Nacional Autónoma de México, Av. Universidad 2001, Cuernavaca, Morelos 62210, Mexico; alagon@ibt.unam.mx; 3 Venom Evolution Lab, School of Biological Sciences, University of Queensland, St. Lucia, Queensland 4067, Australia; rharris2727@googlemail.com; 4 Institute for Molecular Biosciences, University of Queensland, St Lucia, QLD 4072, Australia; Andreas.brust@iinet.net.au (A.B.); p.alewood@imb.uq.edu.au (P.F.A.)

**Keywords:** vasodilatation, potassium channels, *Desmodus rotundus*, vampire bat, venom, calcitonin gene-related peptide

## Abstract

Animals that specialise in blood feeding have particular challenges in obtaining their meal, whereby they impair blood hemostasis by promoting anticoagulation and vasodilation in order to facilitate feeding. These convergent selection pressures have been studied in a number of lineages, ranging from fleas to leeches. However, the vampire bat (*Desmondus rotundus*) is unstudied in regards to potential vasodilatory mechanisms of their feeding secretions (which are a type of venom). This is despite the intense investigations of their anticoagulant properties which have demonstrated that *D. rotundus* venom contains strong anticoagulant and proteolytic activities which delay the formation of blood clots and interfere with the blood coagulation cascade. In this study, we identified and tested a compound from *D. rotundus* venom that is similar in size and amino acid sequence to human calcitonin gene-related peptide (CGRP) which has potent vasodilatory properties. We found that the vampire bat-derived form of CGRP (i.e., vCGRP) selectively caused endothelium-independent relaxation of pre-contracted rat small mesenteric arteries. The vasorelaxant efficacy and potency of vCGRP were similar to that of CGRP, in activating CGRP receptors and Kv channels to relax arteriole smooth muscle, which would facilitate blood meal feeding by promoting continual blood flow. Our results provide, for the first time, a detailed investigation into the identification and function of a vasodilatory peptide found in *D. rotundus* venom, which provides a basis in understanding the convergent pathways and selectivity of hematophagous venoms. These unique peptides also show excellent drug design and development potential, thus highlighting the social and economic value of venomous animals.

## 1. Introduction

Common vampire bats (*Desmondus rotundus*) are found in Central and South America, and feed exclusively on mammalian blood [1,2]. They preferentially feed on livestock animals such as cattle [3] and produce venom components that disrupt the blood coagulation cascade, enabling a constant blood flow for feeding [4,5,6,7]. However, there are reports of rare incidents of human interactions which have led vampire bats to become more medically relevant to humans [8,9]. Outbreaks of rabies in human populations due to the vampire bats being vectors of the disease [10], have led to anti-vampire bat campaigns and culling of bat populations [11,12].

Previous studies have demonstrated that *D. rotundus* venom contains two important anticoagulant toxins: Draculin [6,7,13]; and DSPA (*Desmodus rotundas* salivary plasminogen activator) [14,15]. Draculin is a glycoprotein that irreversibly binds to factors IXa and X, and inhibits the conversion of prothrombin to thrombin [6,7,13]. This prevents fibrinogen being converted into fibrin and thus inhibits coagulation of blood during feeding [5]. DSPA components also aid in ensuring continuous blood flow by breaking up the fibrin mesh of any blood clots that are formed [16]. While there are relatively extensive studies on Draculin and DSPA, little is known about the other components of *D. rotundus* venom, with vasodilation a predicted but untested activity [15,16].

Other hematophagous animals induce anticoagulant and vasodilatory effects through the delivery of bioactive compounds, thus ensuring efficient blood flow for feeding. For example, mosquitos possess tachykinin-like peptides (sialokinins) [17,18], whilst bedbugs possess nitrosyl-hemoproteins (nitrophorins) [19,20]. In addition, sand flies contain a potent vasodilator (maxadilan) that acts via the PAC1 receptor [21,22], and horse fly disintegrins inhibit platelet aggregation like those from snake venoms [23]. Interestingly, tick prostaglandins constrict blood vessels [24]. The maintenance of blood flow during feeding is a major rate limiting step and challenge for blood feeders to overcome. Therefore, the longer they take to feed, the higher the chances the host or prey will notice, making them more vulnerable [25]. Thus, due to the similarities in feeding mechanisms between hematophagous animals, it has been postulated that vasodilators may play a key role in the venom of *D. rotundus*, targeting skin capillaries, to complement coagulation inhibition [15,16]. 

**Figure 1:**
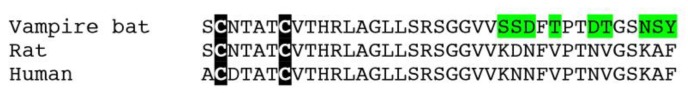
Alignments of vCGRP (Vampire bat), rCGRP (Rat), and hCGRP (human) with cysteines shaded in black and vampire bat specific modified residues in green.

However, such actions have remained speculative until the current study which demonstrated selective and potent action for resistance-like arteries. Previously we showed that the transcriptome and proteinaceous products of the *D. rotundus* hematophagous secretion glands are rich in calcitonin gene related peptide variants [26], which are similar in size and amino acid sequences to CGRP but with modifications in key residues (Figure 1). CGRP is a potent vasodilator that acts via activation of CGRP1 receptors on either endothelial or smooth muscle cells [27,28,29,30]. The significance of this peptide type in relation to the obtaining of blood-meals, and the impact of residues, was tested in order to ascertain the role in securing blood-meals by *D. rotundus.* In this study, we have demonstrated that vCGRP also causes vasodilation of resistance-like arteries via similar pathways to CGRP but with greater selectivity.

## 2. Results

### 2.1. Vasorelaxant Responses to D. rotundus vCGRP and rCGRP

**Figure 2:**
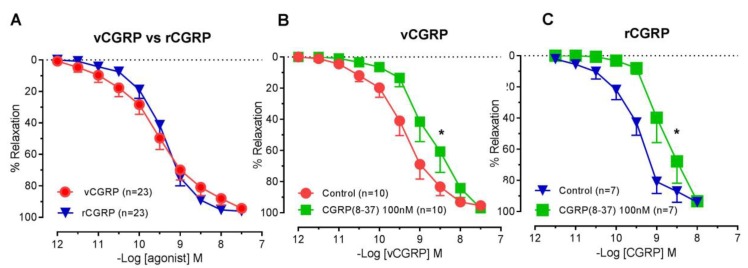
*D. rotundus* vCGRP causes vasodilation similar to rCGRP via CGRP1 receptors. Cumulative concentration-response curves to (**A**) *D. rotundus* vCGRP (*n* = 23) and rat CGRP (*n* = 23) alone and (**B**) *D. rotundus* vCGRP (*n* = 10) and (**C**) rat CGRP (*n* = 7) in the absence and presence of CGRP8-37 (100 nM, *n* = 7–10) in rat small mesenteric arteries. Values are expressed as % reversal of pre-contraction and given as mean ± SEM, where *n* = number of animals. * *p* < 0.05 pEC50 versus control, student’s unpaired *t*-test.

In rat small mesenteric arteries, *D. rotundus* vCGRP was a potent vasorelaxant (pEC_50_ = 9.47 ± 0.32 −logM, R_max_ = 94.6 ± 2.4%) with a potency and efficacy similar to that of rat calcitonin gene-related peptide (rCGRP; pEC_50_ = 9.16 ± 0.17 -logM R_max_ = 93.8 ± 2.6; Figure 2A). In the presence of the rat CGRP1 receptor antagonist CGRP8-37, the potency of *D. rotundus* vCGRP (Figure 2B) and rCGRP (Figure 1C) was decreased by 6-fold (*p* < 0.05) and 5-fold (*p* < 0.05) respectively, with no change in R_max_ (Figure 2B).

### 2.2. Contribution of NO-SGC and Adenylate Cyclase to D. rotundus vCGRP and rCGRP Mediated Relaxation

**Figure 3:**
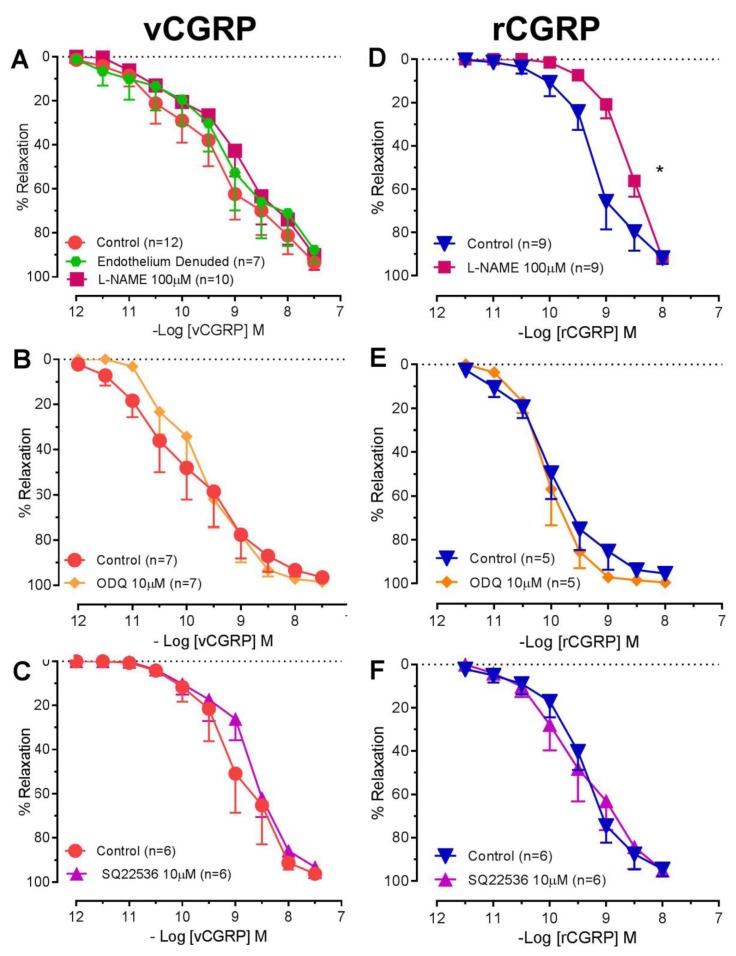
The soluble guanylyl cyclase or adenylyl cyclase pathways do not play a role in vasorelaxation induced by *D. rotundus* vCGRP or rCGRP. Cumulative concentration-response curves to *D. rotundus* vCGRP (**A**–**C**) or rat CGRP (**D**–**F**) in rat small mesenteric arteries in the absence (*D. rotundus* vCGRP, *n* = 6–12; rat CGRP, *n* = 5−9) or presence of either L-NAME (100 µM, *n* = 9–10), ODQ (10 µM, *n* = 5–7), SQ22536 (10 µM, *n* = 6) or following endothelial denudation (*n* = 7). Values are expressed as % reversal of pre-contraction and given as mean ± SEM, where *n* = number of animals. * *p* < 0.05 pEC50 versus control, student’s unpaired *t*-test.

Vasorelaxation to *D. rotundus* vCGRP was unchanged following endothelial denudation or treatment with L-NAME (100 μM) (Figure 3A). In contrast, potency to rCGRP was decreased 5-fold in the presence of L-NAME (100 μM) from 9.16 ± 0.17 to 8.62 ± 0.09 (pEC_50_ = 0.01) with no difference in maximum relaxation (Figure 3D). The presence of the soluble guanylyl cyclase inhibitor ODQ (10 μM) or the adenylyl cyclase inhibitor SQ22536 (10 μM) (Figure 3B,C,E,F) had no significant effect on *D. rotundus* vCGRP or rCGRP relaxation curves. 

### 2.3. Contribution of Potassium Channels to D. rotundus vCGRP and rCGRP Mediated Relaxation

**Figure 4:**
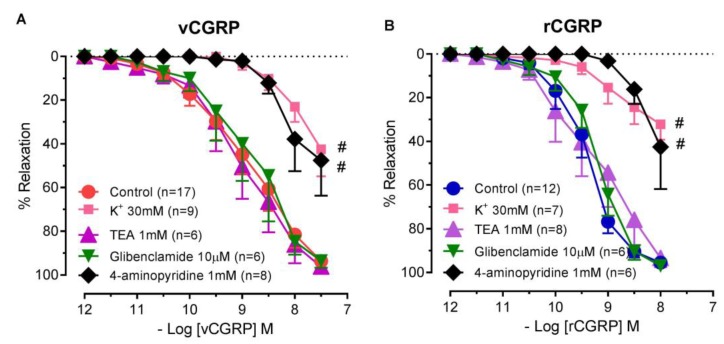
Voltage-gated potassium channels significantly attenuate the vasodilatory effects of *D. rotundus* vCGRP and rCGRP. Cumulative concentration-response curves to (**A**) *D. rotundus* vCGRP (*n* = 17) or (**B**) rat CGRP (*n* = 12) in rat small mesenteric arteries from rats in the absence or presence of either 30 mM K^+^ (*n* = 7–9), TEA (1 mM, *n* = 6−8), glibenclamide (10 µM, *n* = 6) or 4-aminopyridine (1 mM, *n* = 6–8). Values are expressed as % reversal of pre-contraction and given as mean ± SEM, where *n* = number of animals. * *p* < 0.05, concentration-response curve significantly different as compared to control (2-Way ANOVA). # *p* < 0.05, response at 30 nM or 10 nM significantly different as compared to control (1-Way ANOVA, Bonferroni’s post hoc).

Raising the extracellular concentration of K^+^ to 30 mM markedly attenuated the relaxant response to *D. rotundus* vCGRP (Figure 4A). Blocking voltage-dependent K^+^ channels with 4-aminopyridine (1 mM) markedly attenuated *D. rotundus* vCGRP-induced relaxation, reducing the potency by approximately 30-fold (*p* < 0.05) and reducing the response at 10 nM to 53.7 ± 17.3% (*p* < 0.01). However, vasorelaxation to *D. rotundus* vCGRP was unchanged in the presence of the ATP-sensitive K^+^ channel inhibitor, glibenclamide (10 μM), or the Ca^2+^ activated K^+^ channel inhibitor, TEA (1 mM). Similarly, vasorelaxation to rCGRP was attenuated in the presence of 30 mM K^+^ or 4-aminopyridine (1 mM) yet unchanged in the presence of TEA (1 mM) or glibenclamide (10 μM) (Figure 4B).

## 3. Discussion

*D. rotundus* venom is well known to contain anticoagulating properties in order to facilitate blood feeding [26]. Indeed, a glycoprotein, Draculin, which inhibits activated coagulation factors IX (IXa) and X (Xa) has been isolated from *D. rotundus* venom [5]. In the current study, we isolated and characterised a peptide (vCGRP) from the venom, which is similar in size and amino acid sequence to CGRP found in humans and rats. CGRP is a potent vasodilator that acts via activation of CGRP1 receptors on either endothelial or smooth muscle cells [27,31]. Therefore, the aim of this study was to determine whether vCGRP also causes vasodilation via similar pathways. 

We identified vCGRP as a dilator of rat small mesenteric arteries with a potency and efficacy similar to rCGRP. Importantly, like rCGRP, the vasorelaxation was attenuated by the CGRP1 receptor antagonist, CGRP8-37, indicative of an ability of the peptide to target this receptor to mediate its response though direct activation of CGRP1 receptors can be further supported by radioactive ligand binding assays in the future. Next we examined the role of endothelial cells in vasorelaxation via vCGRP. Given the vasorelaxation to vCGRP was unchanged following endothelial denudation or inhibition of nitric oxide synthase (by L-NAME), it is likely that vCGRP targets CGRP1 on vascular smooth muscle cells (VSMC) to cause endothelium-independent relaxation. In contrast, relaxation to rCGRP appeared to be, in part, dependent on endothelial-derived nitric oxide (NO) as the potency was attenuated following NOS inhibition. These findings highlight a potential point of difference with regard to CGRP derived from distinct species. Thus whilst an endothelium-dependent component of vasorelaxation to rCGRP has been observed in mesenteric [32] and retinal [33] arteries, we have demonstrated that vCGRP, like human CGRP [34], mediates relaxation via endothelium-independent mechanisms. This similarity in mechanism of action between human CGRP and vCGRP supports the notion of vCGRP becoming a potential candidate for therapeutic drug discovery.

Previous studies have also demonstrated that activation of CGRP receptors can lead to the activation of the guanylyl cyclase pathway (endothelium-dependent) or adenylyl cyclase pathway (endothelium-independent) [33,34,35,36,37,38]. However, the presence of ODQ (guanylyl cyclase inhibitor) or SQ22536 (adenylyl cyclase inhibitor), had no significant effect on rCGRP or *D. rotundus* vCGRP relaxation curves. Differences between CGRP endothelium-independent and -dependent mechanisms are related to the region, size of the vessel tested and species of CGRP. For instance, human or rat CGRP tested in pig coronary leads to increased cAMP and causes vasorelaxation via endothelium-independent pathways [34]. However, human CGRP tested in human vessels are endothelium-dependent [28]. 

Therefore, we next sought to characterise the mechanism(s) via which vCGRP mediates endothelium-independent relaxation. Our finding that raising the extracellular K^+^ concentration to 30 mM markedly attenuated the relaxation to vCGRP suggests that the peptide modulates relaxation of rat small mesenteric arteries in part via activation of K^+^ channels. Indeed, we identified an ability of vCGRP to activate voltage-dependent K^+^ channels as relaxation responses were decreased by 4-AP. This was in agreement to findings with respect to rCGRP. Neither K_ATP_ nor K_Ca_ channels appeared to be involved in relaxation to vCGRP or rCGRP as glibenclamide and TEA were without effect. Indeed, there is evidence that activation of CGRP receptors could lead to direct opening of K^+^ channels, in particular Kv channels [33]. There are conflicting reports on the involvement of K_ATP_ and K_Ca_ channels in vasorelaxation, which could be related to the type of vessel studied. For instance, studies using bovine retinal arteries and rabbit mesenteric arteries report that activation of K_ATP_ channels, but not K_Ca_ channels, leads to vasorelaxation [37,39,40]. However, studies in smooth muscle cells from rat mesenteric arteries have shown CGRP directly activates BK_Ca_ channels [41]. These data further highlight that CGRP causes vasorelaxation through a variety of mechanisms which is dependent upon the species and vessel involved.

Considering the medical relevance to humans of *D. rotundus* and other vampire bat species as disease vectors for rabies [1], it is surprising that more in depth studies have not been conducted on the intricate mechanisms employed in their feeding behaviour, despite studies on other blood feeding animals such as fleas and leeches [13,26,42]. Such secretions fit within the definition of venom as ‘A secretion produced in specialized cells in one animal, delivered to a target animal through the infliction of a wound and that disrupts endophysiological or biochemical processes in the receiving animal to facilitate feeding, defense or competition by/of the producing animal’ [42]. As peptides used by venoms/hematophagous-secretions are modified versions of those routinely expressed in other tissues [43] future work including the other two species of vampire bat and non-hematophagous bats would be enlightening in regards to the timing of the recruitment for use in blood-feeding and the molecular diversification events. This study has opened the way for further research to investigate the pathways and intricate mechanisms of hematophagous venoms, in particular vampire bats. Therefore, we have made clear the ability of vCGRP to selectively mediate endothelium-independent vasorelaxation in part via activation of Kv channels.

This selectivity of vCGRP to target only vascular smooth cells (similar to that of human CGRP) highlights the interesting possibility that vCGRP may confer benefit in the context of cardiovascular diseases such as hypertension, heart failure and kidney diseases [44]. Further functional studies are required for vCGRP to become a therapeutic intervention with potential pharmacological applications. This research also paves the way for further evolutionary studies into hematophagous venoms.

## 4. Materials and Methods 

Synthesis of vCGRP was accomplished using protocols previously described by us for other peptides [45].

### 4.1. Isolation of Rat Small Mesenteric Arteries

Male Sprague-Dawley rats (200–250 g) were euthanized via CO_2_ inhalation (95% CO_2_, 5% O_2_) followed by exsanguination. Small mesenteric arteries (second-order branch of the superior mesenteric artery) were isolated, cut into 2 mm lengths, and mounted on 40 µm wires in small vessel myographs [46]. Vessels were maintained in physiological salt solution [composed of (in mM) 119 NaCl, 4.7 KCl, 1.17 MgSO_4_, 25 NaHCO_3_, 1.8 KH_2_PO_4_, 2.5 CaCl_2_, 11 glucose, and 0.026 EDTA] at 37 °C and were bubbled with carbogen (95% O_2_, 5% CO_2_). In a subset of arteries, the endothelium was gently denuded via insertion of a 40 µm wire inside the lumen and rubbing the vessel walls. The mesenteric arteries were allowed to equilibrate for 30 min under zero force and then a 5 mN resting tension was applied. Changes in isometric tension were recorded using Myograph Interface Model 610 M version 2.2 (DMT, Aarhus, Denmark) and PowerLab/835 (ADInstruments Inc, Bella Vista, NSW, Australia). Data was recorded with the data acquisition program Chart (V5, ADInstruments). Following a 30 min equilibration period at 5 mN, the mesenteric arteries were contracted maximally (F_max_) using a K^+^ depolarizing solution [K^+^−containing physiological salt solution (KPSS); composed of (in mM) 123 KCl, 1.17 MgSO_4_, 1.18 KH_2_PO_4_, 2.5 CaCl_2_, 25 NaHCO_3_, and 11 glucose]. The integrity of the endothelium was confirmed by relaxation to acetylcholine (ACh, 10 µM) [46] in tissues pre-contracted with the thromboxane A_2_ mimetic, U46619 (1 µM) [46]. Arteries were washed with physiological salt solution and the tension allowed to return to baseline.

### 4.2. Vasorelaxation Experiments

Cumulative concentration-response curves to *D. rotundus* vCGRP (10^−12^–3 × 10^−8^ M) or rCGRP (3 × 10^−12^–10^−8^ M) [32,34,37] were constructed in vessels pre-contracted submaximally (~50% Fmax) with titrated concentration of U46619 (0.01 µM–0.2 µM). Responses to *D. rotundus* vCGRP and rCGRP were obtained in endothelium-intact mesenteric arteries in the absence or presence of either ODQ (10 µM) [47], SQ22536 (10 μM) [48], L-NAME (0.1 µM) [46], CGRP8-37 (0.1 µM) [49,50], 30mM K^+^ [33], TEA (1000 µM), 4-aminopyridine (1000 µM) [46] or glibenclamide (10 µM) [32,51]. All treatments were added for 30 min prior to precontraction with U46619. In a subset of endothelium-denuded arteries, vasorelaxation to *D. rotundus* vCGRP was also examined. Sodium nitroprusside (SNP; 10 µM) [47] was added at the end of each concentration-response curve to ensure maximum relaxation. Only one concentration-response curve to *D. rotundus* vCGRP or rCGRP was obtained in each vessel segment [47,52].

### 4.3. Data Analysis and Statistical Procedures

Relaxation responses were expressed as a percentage reversal of the U46619 pre-contraction. Individual relaxation curves were fitted to a sigmoidal logistic equation and pEC_50_ values (concentration of agonist resulting in a 50% relaxation) calculated and expressed as –Log mol.L^−1^. Statistical comparisons between the experimental groups’ mean pEC_50_ and maximum relaxation (R_max_) values were made using a Student’s unpaired *t*-test or one-way ANOVA with Bonferroni’s post hoc comparison. Where pEC_50_ values could not be obtained, concentration-response curves were compared by means of a two-way ANOVA. *n* = number of artery segments from separate animals. Data represent the mean ± SEM (error bars on graph). Statistical significance was defined as * *p* < 0.05. All data analysis was performed using GraphPad Prism version 5.02 (GraphPad Software, San Diego, CA, USA, 2009) [46].

### 4.4. Reagents

Reagents and their sources were U46619 (Cayman Chemical company, Ann Arbor, Michigan, USA), SQ22356 (Tocris bioscience, Bristol, UK), ODQ, Glibenclamide, TEA, 4-aminopyridine, L-NAME, SNP, ACh, CGRP8-37 (Sigma-Aldrich, St Louis, MO, USA), and CGRP (rat) Peptide Institute, Osaka, Japan. Stock solutions of ODQ (10 mmol/L) and U46619 (1 mM) were dissolved in absolute ethanol. All subsequent dilutions of stock solutions were in distilled water. All other drugs were made up in distilled water and all dilutions were prepared fresh daily.

## References

[1] Greenhall A.M. (2018). Natural History of Vampire Bats.

[2] Casewell N.R., Wüster W., Vonk F.J., Harrison R.A., Fry B.G. (2013). Complex cocktails: The evolutionary novelty of venoms. Trends Ecol. Evol..

[3] Delpietro H., Marchevsky N., Simonetti E. (1992). Relative population densities and predation of the common vampire bat (*Desmodus rotundus*) in natural and cattle-raising areas in north-east argentina. Prev. Vet. Med..

[4] Disanto P.E. (1960). Anatomy and histochemistry of the salivary glands of the vampire bat, *Desmodus rotundus murinus*. J. Morphol..

[5] Fernandez A.Z., Tablante A., Bartoli F., Beguin S., Hemker H., Apitz-Castro R. (1998). Expression of biological activity of draculin, the anticoagulant factor from vampire bat saliva, is strictly dependent on the appropriate glycosylation of the native molecule. Biochim. Biophys. Acta Gen. Subj..

[6] Fernandez A.Z., Tablante A., Beguín S., Hemker H.C., Apitz-Castro R. (1999). Draculin, the anticoagulant factor in vampire bat saliva, is a tight-binding, noncompetitive inhibitor of activated factor X. Biochim. Biophys. Acta Protein Struct. Mol. Enzym..

[7] Apitz-Castro R., Beguin S., Tablante A., Bartoli F., Holt J., Hemker H. (1995). Purification and partial characterization of draculin, the anticoagulant factor present in the saliva of vampire bats (*Desmodus rotundus*). Thromb. Haemost..

[8] Batista-da-Costa M., Bonito R.F., Nishioka S. (1993). An outbreak of vampire bat bite in a Brazilian village. Trop. Med. Parasitol..

[9] Schneider M.C., Aron J., Santos-Burgoa C., Uieda W., Ruiz-Velazco S. (2001). Common vampire bat attacks on humans in a village of the amazon region of Brazil. Cadernos Saúde Pública.

[10] Schneider M.C., Romijn P.C., Uieda W., Tamayo H., Silva D.F.D., Belotto A., Silva J.B.d., Leanes L.F. (2009). Rabies transmitted by vampire bats to humans: An emerging zoonotic disease in Latin America?. Revista Panamericana Salud Pública.

[11] McCarthy T.J. (1989). Human depredation by vampire bats (*Desmodus rotundus*) following a hog cholera campaign. Am. J. Trop. Med. Hyg..

[12] Constantine D.G. (1970). Bats in Relation to the Health Welfare and Economy of Man.

[13] Basanova A., Baskova I., Zavalova L. (2002). Vascular–platelet and plasma hemostasis regulators from bloodsucking animals. Biochemistry.

[14] Tellgren-Roth Å., Dittmar K., Massey S.E., Kemi C., Tellgren-Roth C., Savolainen P., Lyons L.A., Liberles D.A. (2009). Keeping the blood flowing—Plasminogen activator genes and feeding behavior in vampire bats. Naturwissenschaften.

[15] Hawkey C. (1966). Plasminogen activator in saliva of the vampire bat *Desmodus rotundus*. Nature.

[16] Hawkey C. (1967). Inhibitor of platelet aggregation present in saliva of the vampire bat *Desmodus rotundus*. Br. J. Haematol..

[17] Champagne D.E., Ribeiro J. (1994). Sialokinin I and II: Vasodilatory tachykinins from the yellow fever mosquito *Aedes aegypti*. Proc. Nat. Acad. Sci. USA.

[18] Ribeiro J.M., Nussenzveig R.H., Tortorella G. (1994). Salivary vasodilators of *Aedes triseriatus* and *Anopheles gambiae* (Diptera: Culicidae). J. Med. Èntomol..

[19] Nussenzveig R.H., Bentley D.L., Ribeiro J. (1995). Nitric oxide loading of the salivary nitric-oxide-carrying hemoproteins (nitrophorins) in the blood-sucking bug *Rhodnius prolixus*. J. Exp. Boil..

[20] Valenzuela J.G., Walker F., Ribeiro J. (1995). A salivary nitrophorin (nitric-oxide-carrying hemoprotein) in the bedbug *Cimex lectularius*. J. Exp. Boil..

[21] Lerner E.A., Iuga A.O., Reddy V.B. (2007). Maxadilan, a PAC1 receptor agonist from sand flies. Peptides.

[22] Lerner E., Ribeiro J., Nelson R.J., Lerner M. (1991). Isolation of maxadilan, a potent vasodilatory peptide from the salivary glands of the sand fly *Lutzomyia longipalpis*. J. Boil. Chem..

[23] Ma D., Xu X., An S., Liu H., Yang X., Andersen J.F., Wang Y., Tokumasu F., Ribeiro J.M., Francischetti I.M. (2011). A novel family of RGD-containing disintegrin (Tablysin-15) from the salivary gland of the horsefly *Tabanus yao* targets integrins α_IIb_β_3_ and α_v_β_3_ and inhibits platelet aggregation and angiogenesis. Thromb. Haemost..

[24] Tu A.T., Motoyashiki T., Azimov D.A. (2005). Bioactive compounds in tick and mite venoms (saliva). Toxin Rev..

[25] Greenhall A.M. (1988). Feeding behavior. Nat. Hist. Vampire Bats.

[26] Low D.H.W., Sunagar K., Undheim E.A.B., Ali S.A., Alagon A.C., Ruder T., Jackson T.N.W., Pineda Gonzalez S., King G.F., Jones A. (2013). Dracula’s children: Molecular evolution of vampire bat venom. J. Proteom..

[27] Kawasaki H., Takasaki K., Saito A., Goto K. (1988). Calcitonin gene-related peptide acts as a novel vasodilator neurotransmitter in mesenteric resistance vessels of the rat. Nature.

[28] Thom S.M., Hughes A.D., Goldberg P., Martin G., Schachter M., Sever P.S. (1987). The actions of calcitonin gene related peptide and vasoactive intestinal peptide as vasodilators in man in vivo and in vitro. Br. J. Clin. Pharmacol..

[29] Steenbergh P., Höppener J., Zandberg J., Visser A., Lips C., Jansz H. (1986). Structure and expression of the human calcitonin/CGRP genes. FEBS Lett..

[30] Rosenfeld M.G., Mermod J.-J., Amara S.G., Swanson L.W., Sawchenko P.E., Rivier J., Vale W.W., Evans R.M. (1983). Production of a novel neuropeptide encoded by the calcitonin gene via tissue-specific RNA processing. Nature.

[31] Russell F.A., King R., Smillie S.-J., Kodji X., Brain S.D. (2014). Calcitonin gene-related peptide: Physiology and pathophysiology. Physiol. Rev..

[32] Lei S., Mulvany M.J., Nyborg N.C.B. (1994). Characterization of the CGRP receptor and mechanisms of action in rat mesenteric small arteries. Pharmacol. Toxicol..

[33] Boussery K., Delaey C., Van de Voorde J. (2005). The vasorelaxing effect of CGRP and natriuretic peptides in isolated bovine retinal arteries. Investig. Ophthalmol. Vis. Sci..

[34] Shoji T., Ishihara I., Ishikawa T., Saito A., Goto K. (1987). Vasodilating effects of human and rat calcitonin gene-related peptides in isolated porcine coronary arteries. Naunyn-Schmiedebergs Arch. Pharmacol..

[35] Brain S.D., Grant A.D. (2004). Vascular actions of calcitonin gene-related peptide and adrenomedullin. Physiol. Rev..

[36] Gray D.W., Marshall I. (1992). Human α-calcitonin gene-related peptide stimulates adenylate cyclase and guanylate cyclase and relaxes rat thoracic aorta by releasing nitric oxide. Br. J. Pharmacol..

[37] McNeish A.J., Roux B.T., Aylett S.B., Van Den Brink A.M., Cottrell G.S. (2012). Endosomal proteolysis regulates calcitonin gene-related peptide responses in mesenteric arteries. Br. J. Pharmacol..

[38] Zygmunt P.M., Ryman T., HÖGestÄTt E.D. (1995). Regional differences in endothelium-dependent relaxation in the rat: Contribution of nitric oxide and nitric oxide-independent mechanisms. Acta Physiol. Scand..

[39] Nelson M.T., Huang Y., Brayden J.E., Hescheler J., Standen N.B. (1990). Arterial dilations in response to calcitonin gene-related peptide involve activation of K^+^ channels. Nature.

[40] Quayle J.M., Bonev A.D., Brayden J.E., Nelson M.T. (1994). Calcitonin gene-related peptide activated ATP-sensitive K^+^ currents in rabbit arterial smooth muscle via protein kinase A. J. Physiol..

[41] Bol M., Leybaert L., Vanheel B. (2012). Influence of methanandamide and CGRP on potassium currents in smooth muscle cells of small mesenteric arteries. Pflügers Arch. Eur. J. Physiol..

[42] Fry B.G., Roelants K., Champagne D.E., Scheib H., Tyndall J.D., King G.F., Nevalainen T.J., Norman J.A., Lewis R.J., Norton R.S. (2009). The toxicogenomic multiverse: Convergent recruitment of proteins into animal venoms. Annu. Rev. Genom. Hum. Genet..

[43] Fry B.G. (2005). From genome to “venome”: Molecular origin and evolution of the snake venom proteome inferred from phylogenetic analysis of toxin sequences and related body proteins. Genome Res..

[44] Aubdool A.A., Thakore P., Argunhan F., Smillie S.-J., Schnelle M., Srivastava S., Alawi K.M., Wilde E., Mitchell J., Farrell-Dillon K. (2017). A novel α-calcitonin gene-related peptide analogue protects against end-organ damage in experimental hypertension, cardiac hypertrophy, and heart failureclinical perspective. Circulation.

[45] Ruder T., Ali S.A., Ormerod K., Brust A., Roymanchadi M.-L., Ventura S., Undheim E.A., Jackson T.N., Mercier A.J., King G.F. (2013). Functional characterization on invertebrate and vertebrate tissues of tachykinin peptides from octopus venoms. Peptides.

[46] Andrews K.L., Irvine J.C., Tare M., Apostolopoulos J., Favaloro J.L., Triggle C.R., Kemp-Harper B.K. (2009). A role for nitroxyl (HNO) as an endothelium-derived relaxing and hyperpolarizing factor in resistance arteries. Br. J. Pharmacol..

[47] Yuill K.H., Yarova P., Kemp-Harper B.K., Garland C.J., Dora K.A. (2011). A novel role for HNO in local and spreading vasodilatation in rat mesenteric resistance arteries. Antioxid. Redox Signal..

[48] Ross G.R., Yallampalli C. (2006). Endothelium-independent relaxation by adrenomedullin in pregnant rat mesenteric artery: Role of camp-dependent protein kinase a and calcium-activated potassium channels. J. Pharmacol. Exp. Ther..

[49] Oliver K., Kane S., Salvatore C., Mallee J., Kinsey A., Koblan K., Keyvan-Fouladi N., Heavens R., Wainwright A., Jacobson M. (2001). Cloning, characterization and central nervous system distribution of receptor activity modifying proteins in the rat. Eur. J. Neurosci..

[50] Rorabaugh B.R., Scofield M.A., Smith D.D., Jeffries W.B., Abel P.W. (2001). Functional calcitonin gene-related peptide subtype 2 receptors in porcine coronary arteries are identified as calcitonin gene-related peptide subtype 1 receptors by radioligand binding and reverse transcription-polymerase chain reaction. J. Pharmacol. Exp. Ther..

[51] Isomoto S., Kondo C., Yamada M., Matsumoto S., Higashiguchi O., Horio Y., Matsuzawa Y., Kurachi Y. (1996). A novel sulfonylurea receptor forms with BIR (Kir6. 2) a smooth muscle type ATP-sensitive K^+^ channel. J. Boil. Chem..

[52] Favaloro J.L., Kemp-Harper B.K. (2009). Redox variants of NO (NO^·^ and HNO) elicit vasorelaxation of resistance arteries via distinct mechanisms. Am. J. Physiol. Heart Circ. Physiol..

